# The improved bioactive n-HA/PA66 cage versus the PEEK cage in anterior cervical fusion: results from a 6-year follow-up and a case-matched study

**DOI:** 10.1186/s12891-022-06081-3

**Published:** 2022-12-21

**Authors:** Zhipeng Deng, Bowen Hu, Xi Yang, Lei Wang, Yueming Song

**Affiliations:** Department of Orthopedics, Orthopedic Research Institute, West China Hospital, Sichuan University, No. 37 Guo Xue Xiang, Chengdu, 610041 Sichuan China

**Keywords:** Nanohydroxyapatite/polyamide 66 (n-HA/PA66) cage, Polyetheretherketone (PEEK) cage, Anterior cervical decompression and fusion (ACDF)

## Abstract

**Background:**

The nanohydroxyapatite/polyamide 66 (n-HA/PA66) cage, a bioactive nonmetal cage, is fabricated in a hollow cylindrical shape and has been widely used for decades with good clinical outcomes for anterior cervical fusion. However, there remain some radiological complications, such as a slightly high subsidence rate. To improve the clinical outcomes, the improved n-HA/PA66 cage now has been developed into a trapezoidal and wedge shape, a better biomechanical shape matching the cervical spine that is similar to that of the PEEK cage. However, there have been no long-term comparisons of the improved n-HA/PA66 cage and PEEK cage in anterior cervical reconstruction.

**Methods:**

Fifty-eight patients who underwent single-level anterior cervical decompression and fusion (ACDF) with the improved n-HA/PA66 cage (n-HA/PA66 group) were matched with patients with the PEEK cage (PEEK group) by clinical presentation, segment, age and sex. All patients underwent a minimum of 6 years of follow-up. The radiographic parameters (cage subsidence, fusion status, cervical lordosis, and segmental sagittal alignment) and clinical parameters (10-point visual analogue scale, Neck Disability Index and Japanese Orthopedic Association scores) from patients were evaluated before surgery, immediately after surgery, and at the latest follow-up.

**Results:**

The n-HA/PA66 and PEEK groups were well matched in terms of clinical presentation, segment, age, and sex at surgery. The n-HA/PA66 and PEEK cages had similar fusion rates at 6 months postoperatively (n-HA/PA66: 58.6% vs. PEEK: 51.7%, *P* = 0.455) and at the last follow-up (n-HA/PA66: 96.6% vs. PEEK: 93.1%, *P* = 0.402). The respective cage subsidence rates in the n-HA/PA66 and PEEK groups were 6.9 and 12.1% (*P* = 0.342). The correction of SA was similar between the groups at the final follow-up (n-HA/PA66: 4.29 ± 1.99 vs. PEEK: 3.99 ± 2.59 *P* = 0.464). There were no significant differences between the two groups in mean cervical lordosis, visual analogue scale scores of the neck and arm, NDI scores, JOA scores or patients’ overall satisfaction at the final follow-up.

**Conclusion:**

After single-level ACDF, the improved n-HA/PA66 cage had similar excellent results in both radiological and clinical outcomes compared with the PEEK cage over 6 years of follow-up. According to these results, the improved n-HA/PA66 cage and the PEEK cage could be comparable for ACDF.

## Introduction

Anterior cervical decompression and fusion (ACDF), first described by Smith and Robison in the 1950s, has been widely used in the treatment of degenerative disease of the cervical spine [1]. This procedure can directly decompress the stenotic spinal canal and neural foramen, restore the cervical spine sequence and stabilize the corresponding surgical segment [2]. Patients with ACDF have experienced alleviated preoperative pain, postoperative improvements in disability and health-related quality of life, and excellent clinical results. Autologous bone grafts (usually harvested from the iliac crest), with a high fusion rate and superior biocompatibility, have been considered the gold standard cage materials. However, the donor site experiences complications such as pain, infection, and bleeding. Therefore, various cage materials have been developed to replace autologous bone grafts, and they have different clinical features.

Metals were the first materials to be used as substitutes for ACDF cages [3]. Among metals, the titanium mesh cage has been widely used due to its excellent support strength [4]. However, metal materials have the disadvantages of stress shielding, a high subsidence rate and X-ray impenetrability [5]. Subsequently, nonmetal interbody fusion devices with a lower elastic modulus have gradually become a better alternative. Since polyetheretherketone (PEEK) was developed in the 1990s, it has become an extremely popular spinal fusion material due to its excellent mechanical properties and radiolucency [6, 7]. Although PEEK cages have achieved satisfactory long-term patient-reported results in ACDF surgery, PEEK is a bioinert material, rather than an ideal cage material. In recent years, developing bioactive materials for interbody devices has been a popular research topic.

**Fig. 1:**
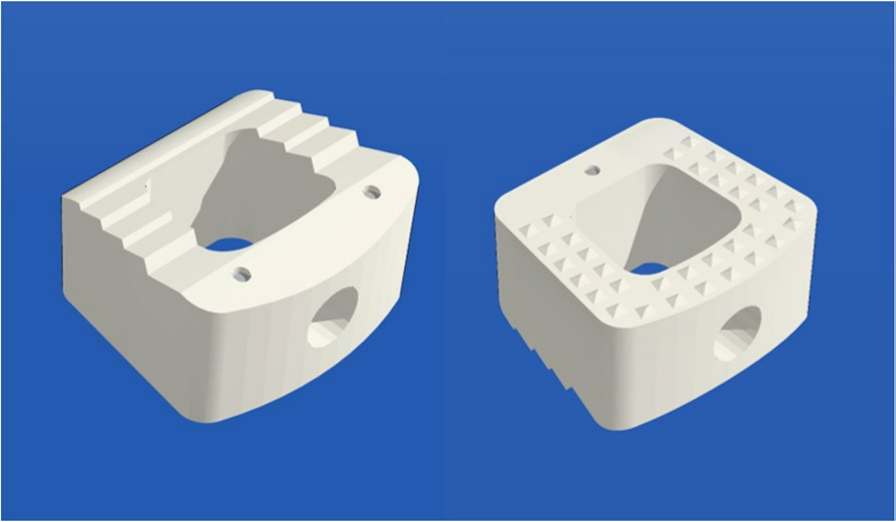
An image of the n-HA/PA66 cage used in the treatment procedure

Nanohydroxyapatite/polyamide 66 (n-HA/PA66) is a synthetic polymer that is biomechanically similar to cortical bone and equally spreads nanohydroxyapatite particles in the PA matrix. It is now an extensively utilized spine bioactive fusion material in clinical because it mimics the structure of human bone tissue and enhances the material’s osteogenic activity [8]. Cages have been fabricated in a hollow cylindrical shape, allowing for bone grafting and growth. It has been reported that the n-HA/PA66 cages provided good stability and a high fusion rate during short- and long-term follow-up following ACDF, comparable to the PEEK cage [9–11]. However, the advantages of bioactive materials were not reflected in these results, and the subsidence rate for the n-HA/PA66 cage was as high as 10.6% [9]. The reason could be that the cylindrical cage had a smaller bone graft volume than that with PEEK and did not distribute vertebral body pressure evenly. To improve the clinical outcomes, the shape of the n-HA/PA66 cage has been developed into a trapezoidal and wedge shape (as shown in Fig. 1), which is a better biomechanical shape with a larger bone graft volume than a hollow cylindrical shape. The trapezoidal cage was retrofitted to the vertebral endplates to increase cage stability during transverse bending, flexion, and axial rotation [12]. And, the wedge cage, a slope design with a higher anterior height, to better restore natural cervical lordosis [13]. This design can mimic the anatomy of the cervical spine, while enhancing local rigidity and increasing surface contact area [14].

Therefore, the purpose of our study was to determine the effectiveness and safety of using the improved bioactive n-HA/PA66 cage for ACDF with anterior fixation in terms of radiological and clinical outcomes compared with those of PEEK.

## Materials and methods

### Patients

The present study was approved by the Institutional Ethics Committee of our hospital. A retrospective review was performed of all patients with cervical degenerative disc disease who received single-level ACDF and anterior fixation using the improved n-HA/PA66 cage in our centre from January 2013 to December 2015. The inclusion criteria were: (1) radiculopathy and/or myelopathy with progressive neurologic deficit; (2) refractory to conservative treatment for at least 4 weeks; (3) involved segment between C3 and C7; and (4) no previous surgical intervention at the cervical spine. The exclusion criteria were tumour(s), tuberculosis, fractures, or infections and patients with insufficient clinical data. Fifty-eight patients (thirty-one male and twenty-seven female) were ultimately enrolled in the present study.

We also reviewed our database to identify patients with degenerative disc disease who had undergone single-level ACDF and anterior fixation using the PEEK cage during the same period and chose those who were the best matches for the patients in the improved n-HA/PA66 group with respect to clinical presentation, segment, age, and sex. If ideal matching was not available for all parameters, the preferred priority was: (1) clinical presentation; (2) segment; (3) age; and (4) sex. Fifty-eight patients (thirty-three male and twenty-five female) served as the PEEK group.

### Surgical procedures

All surgeries were performed in accordance with the established protocols of Smith and Robinson [1]. The overlying cartilage was carefully abraded and removed from the superior and inferior endplates using a high-speed burr and curette. A suitable sized cage was determined in all cases using a trial spacer to confirm the height of the intervertebral disc height at the decompression level; if no suitable original cage was available, the cage was cut to the required size with a saw. Morselized bone from the local decompression was sufficient to fill the cage in all patients. Then, the cage filled with local bone grafts was inserted into the prepared intervertebral space. Each patient received the Atlantis anterior cervical plate system (Medtronic Sofamor Danek, Inc., Memphis, Tennessee, USA). All patients were advised to wear a soft cervical collar for approximately 6 weeks after surgery.

### Radiographic and clinical evaluation

Radiographic outcomes were assessed on cervical plain radiographs and 3-dimensional computed tomographs preoperatively, postoperatively, and after at least 6 years of follow-up. On lateral plain radiographs, the following parameters were assessed: interbody height (IH), segmental lordosis (SA), and cervical sagittal angle (CSA). The IH was measured as the distance between the midpoints of the superior endplate of the upper vertebra and the midpoint of the inferior endplate of the lower vertebra. The difference in height between the postoperative and final follow-up measurements of the fusion segment was defined as the subsidence distance, and a value of more than 2 mm was considered a sign of radiographic subsidence [9, 15]. The SA was defined as the Cobb angle between the superior endplate of the upper vertebral body and the inferior endplate of the lower vertebral body, and the CSA was defined as the Cobb’s angle between the C2 and C7 vertebrae. Moreover, the recovery of the SA (RSA) and CSA (RCSA) was calculated as the difference between the postoperative and preoperative values. All radiographic parameters were assessed by two surgeons who were not involved in the initial operation. The average value of the two measurements was utilized for analysis. Brantigan and Steffee’s grade 5 criteria [16] were utilized by two senior surgeons to assess fusion status using three-dimensional computed tomography scans, with grade 4 or 5 showing fusion.

Clinical outcomes were assessed preoperatively, at 6 months and at the last follow-up postoperatively. Patients were asked to quantify neck and arm pain on a visual analogue scale (VAS) ranging from 0 (no pain/discomfort) to 10 (unbearable pain) pre- and postoperatively. The neck disability index (NDI) questionnaire was used to assess functional results, and the Japanese Orthopedic Association (JOA) score was used to evaluate the neurologic status. Patients’ overall satisfaction with the surgical outcomes was rated using Odom’s criteria [17] at the last follow-up, and the outcomes were deemed satisfactory when they were excellent or good.

### Statistical analysis

Statistical analysis was performed using SPSS software, version 23.0 (SPSS Inc., Chicago, Illinois, USA). Parametric continuous variables are expressed as the means ± standard deviations. Tests of variance between the two groups were conducted using Student’s t test or Wilcoxon’s rank sum test for numerical data. Fisher’s exact test or the X^2^ test was used for categorical variables. *P* < 0.05 was considered a significant difference.

## Results

### General outcomes

All patients underwent single-level ACDF. The n-HA/PA66 and PEEK groups were well matched in terms of clinical presentation, segment, age, and sex when submitted to surgery. There were 58 patients in each group. The mean ages at surgery in the n-HA/PA66 and PEEK groups were 51.9 ± 9.6 years old and 52.4 ± 9.9 years old, respectively (*P* = 0.790). The n-HA/PA66 group had 34 men and 24 women, whereas the PEEK group had 36 men and 22 women (*P* = 0.704). This distribution of the clinical presentation and segments was statistically the same between the groups. The operative levels were C3–C4 in 8 patients (13.8%), C4–C5 in 12 patients (20.7%), C5–C6 in 20 patients (34.5%), and C6–C7 in 18 patients (30.0%). Twenty-one patients (36.2%) presented with radiculopathy, 18 patients (30.0%) presented with myelopathy, and 19 patients (32.8%) presented with radiculomyelopathy, all of which were caused by cervical disc herniation. The mean follow-up period was 87.4 ± 8.2 months in the n-HA/PA66 group and 86.7 ± 7.8 months in the PEEK group. There were no significant differences between the groups in terms of the numbers of smokers and drinkers (Table 1).

**Table 1:** The demographic and clinical data in the two groups

Variables	nHA/PA66 group(*n* = 58)	PEEK group(n = 58)	P
Age	51.9 ± 9.6	52.4 ± 9.9	0.790
Male/Female	34/24	36/22	0.704
Smoker	15	14	0.830
Drinker	11	10	0.809
Follow-up	87.4 ± 8.2	86.7 ± 7.8	0.626
Clinical presentation			
Radiculopathy	21	21	
Myelopathy	18	18	
Radiculomyelopathy	19	19	
Segment			
C3–4	8	8	
C4–5	12	12	
C5–6	20	20	
C6–7	18	18	

### Radiological outcomes

The radiographic data from the surgeries are shown in Table 2. The interbody fusion rate was evaluated by CT scans in all patients at 6 months postoperatively and at the last follow-up. In the nHA/PA66 group, the fusion rate at 6 months postoperatively was 58.6% (34/58), compared with 51.7% (30/58) in the PEEK group (*P* = 0.70). In the n-HA/PA66 group, fusion grade 5 (complete fusion) occurred in 20 patients (34.5%), and fusion grade 4 (probable fusion) occurred in 14 (24.1%); in the PEEK group, the fusion was grade 5 in 16 patients (27.6%) and grade 4 in 14 (24.1%). At the last follow-up, the rates of satisfactory fusion (grade 5 and grade 4) in the n-HA/PA66 and PEEK groups were 96.6 and 93.1%, respectively (*P* = 0.70). Fusion grade 5 was achieved in 43 patients (74.1%) and grade 4 (probable fusion) in 13 (22.4%) in the n-HA/PA66 group; in the PEEK group, there were 37 (63.8%) and 17 (29.3%) patients with fusion grades 5 and 4, respectively. The unfused patients in both groups had asymptomatic grade 3 fusion, and no patients underwent revision surgery.

**Table 2:** Radiographic Outcomes

Variables	nHA/PA66 group	PEEK group	P
Fusion rate (%)			
6 m-Post-O	58.6% (34/58)	51.7% (30/58)	0.455
Final follow-up	96.6% (56/58)	93.1% (54/58)	0.402
IH (mm)			
Preoperatively	35.96 ± 2.52	35.67 ± 2.34	0.520
6 m-Post-O	37.56 ± 2.43	37.35 ± 2.21	0.625
Final follow-up	35.99 ± 2.15	35.73 ± 2.20	0.492
Subsidence (mm)	1.55 ± 0.68	1.62 ± 0.72	0.604
Final subsidence rate	6.9% (4/58)	12.1% (7/58)	0.342
SA			
Preoperatively	1.92 ± 3.50	1.91 ± 3.48	0.985
6 m-Post-O	6.95 ± 3.06	6.76 ± 2.63	0.718
Final follow-up	6.22 ± 3.01	5.89 ± 2.53	0.527
Correction	4.29 ± 1.99	3.99 ± 2.59	0.464
CSA			
Preoperatively	12.69 ± 9.64	11.57 ± 8.88	0.518
6 m-Post-O	21.17 ± 12.28	18.75 ± 10.36	0.255
Final follow-up	20.41 ± 11.06	19.66 ± 11.98	0.748

**Fig. 2:**
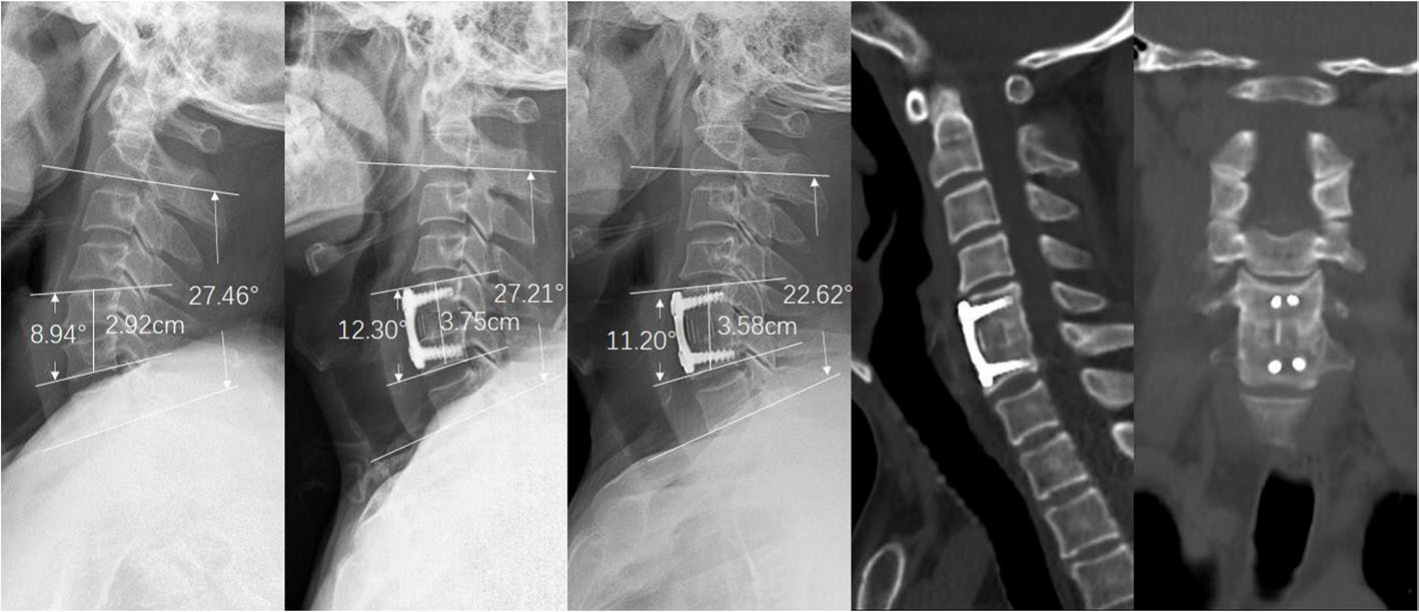
A case of a 50-year-old female patient with cervical myelopathy due to C5/6 disc herniation. **a** Preoperative lateral radiograph of the cervical spine showing narrowing of the disc space between C5/6. **b** Postoperative lateral radiograph shows that the improved n-HA/PA66 cage increased the SA and IH. **c** The 6-year follow-up radiograph showed no subsidence, and the SA remained stable. **d**, **e** Three-dimensional CT at the 6-year follow-up time shows grade 5 fusion. CT, computed tomography; IH, interbody height; n-HA/PA66, nanohydroxyapatite/polyamide66; SA, segmental lordosis

**Fig. 3:**
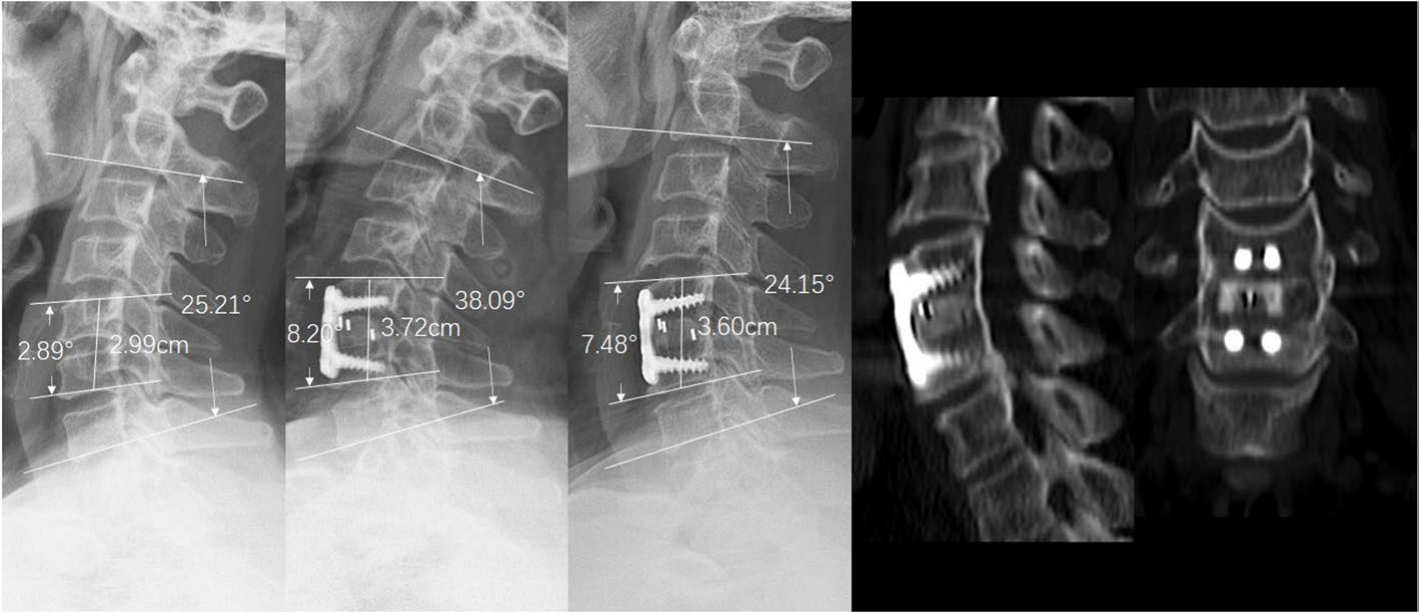
A 49-year-old female patient underwent ACDF with the PEEK cage due to C5/6 disc herniation. **a** Preoperative lateral radiograph of the cervical spine and narrowing of the disc space between C5/6. **b** Postoperative radiograph indicated that the SA and IH improved. **c** The 8-year follow-up radiograph showed stable SA without subsidence. **d**, **e** Three-dimensional CT at the 8-year follow-up showed grade 5 fusion. ACDF, anterior cervical decompression and fusion; SA, segmental lordosis; IH, interbody height; CT, computed tomography; PEEK, polyetheretherketone

The preoperative IH was 35.96 ± 2.52 mm in the n-HA/PA66 group and 35.67 ± 2.34 mm in the PEEK group (*P* = 0.52). After surgery, the IH increased to 37.56 ± 2.43 mm and 37.35 ± 2.21 mm in the two groups, respectively (*P* = 0.625). In both groups, the IH increased significantly postoperatively (*P* = 0.001 and *P* < 0.001). At the final follow-up, the IH decreased to 35.99 ± 2.15 mm and 35.73 ± 2.20 mm, which was not statistically significantly different (*P* = 0.492). The preoperative SA was 1.92 ± 3.50° in the n-HA/PA66 group and 1.91 ± 3.48° in the PEEK group (*P* = 0.985). Postoperatively, the SA increased to 6.95 ± 3.06° in the n-HA/PA66 group and 6.76 ± 2.63° in the PEEK group. Although both groups had significantly increased SAs postoperatively (*P* < 0.001 and P < 0.001), the angles were not significantly different between the two groups (*P* = 0.718). At the final follow-up, the SA decreased to 6.22 ± 3.01° and 5.89 ± 2.53°, respectively (*P* = 0.527). The correction of the SA was similar between the groups at the final follow-up (4.29 ± 1.99 vs. 3.99 ± 2.59 *P* = 0.464). There was no significant difference between the 2 groups in preoperative cervical lordosis (12.69 ± 9.64° vs. 11.57 ± 8.88), and the average cervical lordosis values at the final follow-up in the n-HA/PA66 and PEEK groups were 20.41 ± 11.06 and 19.66 ± 11.98, respectively. Representative patient cases in each group are presented as figures (Figs. 2 and 3).

### Clinical outcomes

In the n-HA/PA66 group, the mean VAS scores of the neck and arm before surgery were 5.17 ± 2.64 and 5.55 ± 2.05, respectively, which significantly improved to 3.24 ± 1.79 and 3.09 ± 1.45, respectively, at 6 months after surgery (*P* < 0.001). In the PEEK group, the mean preoperative VAS scores of the neck and arm were 5.17 ± 2.64 and 5.55 ± 2.05, respectively, and significantly improved to 2.91 ± 1.53 and 3.16 ± 1.31, respectively, 6 months after surgery (P < 0.001). At the last follow-up, neck and arm pain in both groups were further significantly relieved (n-HA/PA66 group: 1.21 ± 0.91 and 1.38 ± 1.15 vs. PEEK group: 1.41 ± 1.01 and 1.28 ± 1.01). The preoperative NID scores of the patients were 32.78 ± 11.25 and 31.48 ± 10.25 in the two groups, respectively (*P* = 0.519). They were improved to 9.34 ± 6.18 in the n-HA/PA66 group and 10.07 ± 6.05 in the PEEK group at the last follow-up (*P* = 0.422). The preoperative JOA scores of the patients were 8.62 ± 1.66 and 8.45 ± 1.50 in the two groups, respectively (*P* = 0.559). They were improved to 14.21 ± 2.12 in the n-HA/PA66 group and 14.31 ± 2.05 in the PEEK group at the last follow-up (P = 0.422). In addition to both groups demonstrating significant improvement in both NID scores (*P* < 0.001) and JOA scores postoperatively (P < 0.001), the recovery rates of the NID and JOA scores were similar. According to the Odom criteria, 50 of 58 patients (86%) were graded excellent to good in the n-HA/PA66 group compared with 52 of 58 patients (90%) in the PEEK group, and there were no statistically significant differences (*P* = 0.879) (Table 3).

**Table 3:** Clinical Outcomes

Variables	nHA/PA66 group	PEEK group	P
VAS-neck			
Preoperatively	5.17 ± 2.64	4.84 ± 2.46	0.473
6 m-Post-O	3.24 ± 1.79	2.91 ± 1.53	0.291
Final follow-up	1.21 ± 0.91	1.41 ± 1.01	0.249
VAS-arm			
Preoperatively	5.55 ± 2.05	5.33 ± 1.86	0.539
6 m-Post-O	3.09 ± 1.45	3.16 ± 1.31	0.789
Final follow-up	1.38 ± 1.15	1.28 ± 1.01	0.607
NDI			
Preoperatively	32.78 ± 11.25	31.48 ± 10.25	0.519
6 m-Post-O	19.71 ± 9.30	18.40 ± 8.05	0.419
Final follow-up	9.34 ± 6.18	10.07 ± 6.05	0.525
NDI recovery rate (%)	69.13 ± 22.22	65.67 ± 24.02	0.422
JOA scores			
Preoperatively	8.62 ± 1.66	8.45 ± 1.50	0.559
6 m-Post-O	13.36 ± 2.25	13.79 ± 2.23	0.302
Final follow-up	14.21 ± 2.12	14.31 ± 2.05	0.790
JOA recovery rate (%)	65.41 ± 26.86	67.83 ± 25.35	0.619
Odom’s criteria			
excellent	15	13	
Good	38	39	
Fair	4	6	
Poor	1	0	
Successful treatment	91.4% (50/58)	90.0% (52/58)	0.879

### Complications

In the n-HA/PA66 group, one patient developed postoperative temporary dysphagia. In the PEEK group, one patient developed cerebrospinal fluid leakage. The complications were resolved after conservative treatment. No major neurological complications occurred in either group of patients. There were no implant-related complications, such as displacement, loosening, breakage, or displacement.

## Discussion

The improved n-HA/PA66 cage has been developed from the original hollow cylindrical shape to a trapezoidal and wedge shape, which has a better biomechanical property. The morphology of the improved n-HA/PA66 cage and PEEK cages is similar, and the difference between them is mainly in the material properties. PEEK is a bioinert material, while n-HA/PA66 is a bioactive material. To study the long-term outcomes of the improved n-HA/PA66 cage and PEEK cage in ACDF, focusing on material differences, only single-segment was included. The retrospective case-matched study showed that excellent radiographic fusion, low subsidence, and great clinical results were essentially similar in patients with improved n-HA/PA66 cages and PEEK cages. Although the improved n-HA/PA66 group had a slightly greater fusion rate and better intervertebral height and segmental angle maintenance on imaging than the PEEK group, these differences were not statistically significant.

An n-HA/PA66 cage composite with an n-HA to PA66 ratio of 6:4 was used as the biomimetic scaffold [18]. The n-HA crystals used in this cage have a composition and structure that is remarkably similar to those of natural bone materials. It can support osteocyte adhesion and proliferation and induce osteocytes to release a range of osteogenic differentiation factors. Moreover, it plays a significant role in osteoconduction and supplies crystal nuclei for the calcification of osteocytes throughout the calcification process. Therefore, it has been considered an ideal material for constructing bone tissue engineering scaffolds [19, 20]. The n-HA crystals impart biological activity to the n-HA/PA66 cage, providing a calcium-phospho-rich microenvironment for bone conduction and induction after implantation [18, 21]. This fact might explain why the n-HA/PA66 cage group had a slightly higher fusion rate at 6 months than the PEEK cage group. However, the difference was not statistically significant, and at the last follow-up, the fusion rates were similar in both groups. The findings were in line with earlier studies [9, 10]. We speculated that there were two main reasons for this unexpected phenomenon. On the one hand, the quantity of autografts is positively correlated with fusion rates [22, 23]. The bone graft volumes in both cages were similar and sufficiently large to be filled with bone fragments during surgery, and they had enough bone mass for osteogenesis. On the other hand, the pore size and porosity played crucial roles in the scaffold architecture and cell proliferation, differentiation and bone in-growth [24]. In the goat C3/4 partial discectomy and fusion model, the mean CT fusion scores of the porous n-HA/PA66 group were significantly higher than those of the dense strut group (porous group vs. dense group: 15.33 ± 2.55 and 10.67 ± 2.55 at 12 weeks, 23.60 ± 3.57 and 16.60 ± 4.67 at 24 weeks, *P* < 0.05). There was a significant difference in the fusion rate between the two groups. Histologic evaluation showed that the mean new bone volumes of the porous n-HA/PA66 group and the dense strut group after surgery were 13.27 ± 2.87 and 42.80 ± 8.56 at 12 weeks and 20.93 ± 3.39 and 68.13 ± 14.03 at 24 weeks, respectively (P < 0.05). As a result, faster bone in-growth occurred with the porous struts [25]. The improved n-HA/PA66 cage is still dense, which could limit its biological activity. To overcome these drawbacks, a novel porous n-HA/PA66 composite has been developed in recent years [24–27]. More effort is necessary to translate it into clinical use, which might enhance radiological and clinical results.

Cage subsidence in ACDF can be influenced by many factors, including end plate preparation, postoperative cervical motion, cage design and material properties, implantation of the anterior cervical plate, and bone mineral density or age [28–31]. This case-matched study was performed to reduce the confounding factors as much as logically and reasonably possible to examine the impact of the subsidence of material characteristics, and the two groups were well matched. Furthermore, the n-HA/PA66 cage was improved to be a more compatible shape with favourable biomechanics, consistent with the PEEK cage. Although the subsidence rate decreased to 6.9% from the previously reported 10.6% with the old cylindrical n-HA/PA66 cage, there was no significant difference compared with the PEEK group (12.1%) [9].

Subsidence is believed to be related to high pressures transferred through interbody spacers on a small surface area. The elastic modulus of the n-HA/PA66 cage was 5.6 GPa, and that of the PEEK cage was 3.5 to 4 GPa [32, 33]. Both cages are similar to natural bone, resulting in lower stress shielding and avoiding some stress shielding accompanied by metallic implants. However, the elastic modulus of the cartilage endplate and cancellous bone (0.1–0.5 GPa) is lower than that of both cages, indicating that subsidence at the interface seems inevitable. In this study, the subsidence of the n-HA/PA66 group was slightly lower than that in the PEEK group, likely because the former fused faster. The more important reason is that the n-HA/PA66 composite has better osseointegration properties and an improved integrated bone-implant interface. Animal experiments have demonstrated that the PEEK cage generates peri-implant inflammatory factors after implantation, gradually forming a fibrous tissue layer on the cage surface that bridges the graft for poor osteogenesis. There was an evident radiolucent rim at the bone graft/PEEK interface with no bone integration, which was called “PEEK-Halo” [34]. The same phenomena were reported by Li et al. who found that the PEEK implant showed a fibrous inert interface and less bone formation, and the PEEK halo line could be seen clearly during long-term observation. In contrast, in the n-HA/PA66 group, these authors discovered a radiolucent gap at the margin of the n-HA/PA66 implant by X-ray radiography and histological sections in the early weeks after implantation (4–8 weeks). Subsequently, the zone decreased and disappeared gradually by 24 weeks. Histological analysis confirmed that more newly formed bone was observed around the n-HA/PA66 implants than PEEK implants during the entire implantation period, and the new bone grew into part of the n-HA/PA66 implant, by which the strut could be integrated with the host bone [35]. Therefore, n-HA/PA66 has better solid anchoring with bone tissue than PEEK, so the intervertebral height and segment angle were better maintained in the n-HA/PA66 group.

In a long-term study, we observed a significant decline in neck and arm pain VAS scores, and the scores did not significantly differ between the two groups at any time point. The NDI and JOA scores showed satisfactory improvement in both groups. No serious complications occurred in our study. The satisfaction rates of the two groups reached those in previous reports [36]. Although one disc undergoing ACDF with n-HA/PA66 cages exhibited solid union on radiographs, the patient complained of neck discomfort, and the clinical results were assessed as poor. Further investigation revealed that the patient suffered from depression pre- and postoperatively. Depression can have a strong association with postoperative outcomes [37]. Therefore, we advocate that surgeons must not only master superb surgical techniques but also relate to the mental health of their patients. Overall, the present result is comparable with our previous results from a series of 98 patients who underwent single-level anterior cervical decompression and fusion (ACDF) using a PEEK cage or an n-HA/PA66 cage for cervical degenerative disease [9].

There were some limitations in the present study. It was a retrospective analysis, and the number of cases was relatively small. Prospective studies with a large number of patients are required to confirm the present findings. This study mainly focused on the comparison of two different cages, and thus the inclusion criteria were relatively strict. And the application in multi-segment ACDF was not discussed. In multi-segmental ACDF, the improved cage has also achieved good outcomes, which will be reported in the future.

## Conclusion

In terms of radiographic fusion, subsidence, segmental angle maintenance and clinical results, the improved bioactive n-HA/PA66 cage had a slight advantage over PEEK in single-level ACDF. However, the difference was not statistically significant. This outcome indicates that the improved n-HA/PA66 cage, which could be comparable with the PEEK cage, is an ideal implant for application in ACDF.

## Data Availability

Data will be available upon request to the first author Zhipeng Deng.
